# Is adding more indicators to a latent class analysis beneficial or detrimental? Results of a Monte-Carlo study

**DOI:** 10.3389/fpsyg.2014.00920

**Published:** 2014-08-21

**Authors:** Ingrid C. Wurpts, Christian Geiser

**Affiliations:** ^1^Department of Psychology, Arizona State UniversityTempe, AZ, USA; ^2^Department of Psychology, Utah State UniversityLogan, UT, USA

**Keywords:** latent class analysis, sample size, number and quality of indicators, covariates of class membership

## Abstract

The purpose of this study was to examine in which way adding more indicators or a covariate influences the performance of latent class analysis (LCA). We varied the sample size (100 ≤ *N* ≤ 2000), number, and quality of binary indicators (between 4 and 12 indicators with conditional response probabilities of [0.3, 0.7], [0.2, 0.8], or [0.1, 0.9]), and the strength of covariate effects (zero, small, medium, large) in a Monte Carlo simulation study of 2- and 3-class models. The results suggested that in general, a larger sample size, more indicators, a higher quality of indicators, and a larger covariate effect lead to more converged and proper replications, as well as fewer boundary parameter estimates and less parameter bias. Furthermore, interactions among these study factors demonstrated how using more or higher quality indicators, as well as larger covariate effect size, could sometimes compensate for small sample size. Including a covariate appeared to be generally beneficial, although the covariate parameters themselves showed relatively large bias. Our results provide useful information for practitioners designing an LCA study in terms of highlighting the factors that lead to better or worse performance of LCA.

## Introduction

Latent class analysis (LCA) is a latent variable modeling technique that identifies latent (unobserved) subgroups of individuals within a population based on nominal or ordinal indicators (Vermunt and Magidson, 2004). LCA is similar to factor analysis in that both methods use one or more latent variables to explain associations among a set of observed variables. Whereas factor analysis clusters observed variables into homogenous groups of indicators, LCA clusters individuals into latent classes. To illustrate, Geiser et al. (2006) applied LCA to identify five subgroups of individuals who used different cognitive strategies to solve mental rotation problems. In another application, LCA classification of eating disorder patients has shown better predictive validity of mortality rates than classifications based on the DSM-IV criteria (Crow et al., 2011).

The use of LCA in applied research has increased dramatically over the past 10 years, as indicated by a search of the PsycInfo database. Whereas in 2002, only 15 articles mentioned LCA, this amount increased to 104 articles in 2013. Despite the widespread interest in and use of LCA in the social sciences, many researchers are still unsure about how different factors influence the performance of LCA. For example, in our experience as statistical consultants, researchers often ask us questions like “Can I apply LCA given my sample size?,” “How many indicators can/should I use in my analysis?,” “Is it better if I use all available indicators or should I use only a subset of indicators given my sample size?,” or “Is adding a covariate to the model beneficial or does it place an additional burden on the estimation?”

Although a few studies have examined issues of sample size (Finch and Bronk, 2011) and indicator quality (Collins and Wugalter, 1992), many practical aspects related to the applicability of LCA are still unclear. This includes the relevance of the number and quality of latent class indicators and the effect of covariates on the quality of parameter estimation in LCA. Moreover, no study to our knowledge has yet examined the interaction among such factors.

High quality indicators are those with strong relationships to the latent class variable (i.e., showing conditional response probabilities close to one or zero). Although using the best quality indicators is ideal, this may not always be possible in practice. Therefore, the question arises, under what conditions can lower quality indicators be used and still produce reliable and unbiased results? In the same vein, can high quality indicators compensate for otherwise suboptimal conditions, such as a small sample size? Is adding more indicators or a covariate generally beneficial or detrimental to the quality of estimation in LCA?

In the present study, we aimed to address the following questions in particular: does the number and quality of latent class indicators as well as the inclusion of a covariate have an influence on the quality of the parameter estimation? Can the use of more and/or higher quality latent class indicators as well as a covariate compensate for the negative effects of smaller sample sizes? We present the findings of a Monte Carlo simulation study in which we explored the impact of these and other conditions on the estimation of LCA parameters. Next, we provide the formal models of LCA and a description of previous methodological research on LCA and mixture models in general.

### The formal models of LCA

In LCA, there are two types of model parameters (e.g., Collins and Lanza, 2010): class proportion parameters and item parameters. Let *L* be the latent class variable with *c* = 1, …, *C* categories (or classes). Then γ_*c*_ = *P*(*L* = *c*) is the unconditional probability of membership in latent class *c*, indicating the proportion of individuals in a particular class, or the relative size of the class. Let *P*(*Y_j_* = *y_j_*|*L* = *c*) be the conditional probability of choosing response *y_j_* for item *j* given membership in class *c* (Vermunt, 2003). This so-called CRP gives the probability of an individual endorsing a specific item category, given the individual is in a certain class. CRPs are particular to each item and class and are used to interpret each class in terms of a characteristic profile of item responses. The complete LCA model for an observed item response pattern **y** out of an array of response patterns **Y** is given by:

(1) $$P\left(Y=y\right)=\sum_{c=1}^{C}P\left(L=c\right)\prod_{j=1}^{J}P\left(Y_{j}=\;y_{j}|L=c\right)$$

The unconditional LCA model shown above can be extended to include covariates for predicting class membership probabilities via a logistic regression model. Given a covariate *X*, the probability of membership in class *c* can be expressed as

(2) $$P\left(L=c|X=x\right)=\frac{e^{\beta_{0c}+\beta_{1c^{x}}}}{\sum_{c=1}^{C}e^{\beta_{0c}+\beta_{1c}x}}$$

In this logistic regression Equation 2, β_0*c*_ is a logistic regression intercept and β_1*c*_ is a logistic regression slope coefficient. We assume that the last class *C* serves as reference class. The regression parameters in the reference class *C* are set to zero (i.e., β_0*C*_ = β_1*C*_ = 0). *e*^β_1*c*_^ represents the odds ratio (OR), or the change in odds of latent class membership between class *c* and the reference class *C* for every one-unit change in *X*. Including a covariate in the model does not change the interpretation of CRPs, but class proportions are now conditional on the value of *X*.

### Previous research on the performance of LCA under different conditions

#### Sample size

Collins and Wugalter (1992) studied the performance of latent transition analysis (LTA), a longitudinal extension of LCA. They found that a “sufficiently large” sample size helped ensure good parameter recovery with few indicators, leading these authors to suggest a minimum *N* of “somewhat smaller than 300” (p. 150), although they only tested *N* = 300 and *N* = 1000 conditions. Tueller and Lubke (2011) examined structural equation mixture models (SEMM; a combination of structural equation and latent class models) and suggested a minimum sample size ranging from *N* = 300 to *N* = 1000. However, the results for SEMM may not apply in the same way to classical LCA. Steinley and Brusco (2011) examined model-based clustering and found that even with *N* = 1000, fit indices (including the Bayesian Information Criterion) only chose the correct number of clusters 42% of the time, suggesting a much higher sample size would be necessary for such fit indices to determine the correct number of clusters. Finch and Bronk (2011) concluded that *N* = 500 is a “worthy goal” (p. 148) for researchers using classical LCA, but did not provide direct empirical evidence for this suggestion.

#### Number of latent class indicators

In general, adding indicators to an LCA model increases the number of possible response patterns, some of which may be observed infrequently. This can lead to data sparseness, low power of chi-square goodness-of-fit tests (Langeheine et al., 1996), and an increase in the number of boundary parameter estimates (Galindo-Garre and Vermunt, 2006). Boundary parameter estimates are probabilities estimated to be exactly zero or one. Some researchers view these as problematic because they imply “perfect reliability” of an indicator, which is unlikely in practice. Also, no standard errors and therefore no confidence limits are available for boundary parameter estimates (Galindo-Garre and Vermunt, 2006). Moreover, their presence can cause numerical problems in estimation algorithms (Vermunt and Magidson, 2004) as well as problems in computing the parameter's asymptotic variance-covariance matrix (Galindo-Garre and Vermunt, 2006). Boundary estimates may also indicate identification problems or the convergence to a local likelihood maximum and may be difficult to interpret (Uebersax, 2000). Thus, boundary parameters can present both statistical and substantive difficulties.

Because potential complications can arise from data sparseness and boundary parameter estimates, researchers may prefer using fewer indicators with LCA. On the other hand, Marsh et al. (1998) found that using more high-quality indicators per latent variable in confirmatory factor analysis (CFA) resulted in several advantages: more converged solutions, more proper solutions, and less positive and negative parameter bias. These advantages were even more pronounced for smaller sample sizes and may have occurred because adding more indicators provides additional information that can be used in the estimation of latent variable parameters. Similarly, Collins and Wugalter (1992) suggested that adding additional indicators to LTA can outweigh the disadvantage of data sparseness by reducing standard errors. Peugh and Fan (2013) found that enumeration indices usually led to an overextraction of latent classes in latent profile analysis (LPA) models with more indicators. Tein et al. (2013) found that in general, LPA models with more indicators had higher power to detect the correct number of classes. However, a more thorough and systematic examination of how the number of indicators specifically affects parameter bias in LCA is still missing.

#### Quality of latent class indicators

High quality indicators are predicted by the latent variable to have a probability near one or zero. Such indicators are generally desirable for model estimation and the interpretation of the latent classes. However, Galindo-Garre and Vermunt (2006) found that indicators with high population values (i.e., conditional response probabilities close to one) were more likely to produce boundary estimates in LCA applications, even though this effect decreased with larger *N*. Still, there is evidence that using high quality indicators is generally beneficial, at least in the context of structural equation models with continuous latent variables (Marsh et al., 1998). Having more high quality indicators can stabilize the estimation by increasing the information available to estimate latent variable parameters. In this vein, Collins and Wugalter (1992) speculated that having sufficiently high quality indicators may compensate for having few indicators and may aid parameter recovery in LTA models.

#### Inclusion of covariates

Even though a large number of LCA applications include one or more covariates to predict class membership, relatively little is known about how covariates influence estimation quality in LCA. Clark and Muthén (2009) showed that the single-step inclusion method performs best at recovering the true covariate parameter effect, and that it has the highest power and coverage of the effect. However, Clark and Muthén's simulation only considered 2-class models with 10 indicators and two different covariate effect sizes. Also, Clark and Muthén's study focused on comparing methods of covariate inclusion rather than on the question of whether covariate inclusion enhances the performance of LCA in general. None of the conditions were compared to similar conditions without a covariate, and potential bias in other model parameters (e.g., class proportion bias, conditional response probability, or CRP bias) was not examined in their study.

Other simulation work has examined the use of covariates in factor mixture models (a combination of LCA and common factor analysis) and found that increasing the covariate effect size leads to a higher proportion of individuals assigned to the correct class, even if class separation is poor (Lubke and Muthén, 2007), and that covariates can aid in correctly determining the number of classes (Muthén, 2004). In using real data to estimate an SEMM with and without covariates, the model with covariates performed better, as determined by the BIC (Vermunt and Magidson, 2005). On the other hand, not much is known about whether the inclusion of covariates can help compensate for small samples, too few indicators, or low quality indicators. This study adds an important contribution to the literature by examining in detail the effects of covariate inclusion simultaneously with sample size and number and quality of indicators.

In summary, few studies have examined the performance of LCA under different conditions. Given the increasing popularity of LCA, it is important for researchers to know which factors and their interactions influence the performance of LCA.

### Hypotheses

Although there have been few simulation studies examining the effects of including covariates in LCA, we expected that a large covariate effect size would improve parameter recovery, and that models with a strong covariate would perform better in general than models without a covariate or with only weak covariate effects. We expected that adding more and higher quality indicators would be beneficial and would allow (to some extent) the use of smaller samples. High quality indicators were expected to improve parameter recovery, but also increase boundary parameter estimates, at least in small sample sizes. In line with previous research, we expected that *N* ≥ 500 would consistently perform well and that conditions below *N* = 300 may be problematic.

## Methods

### Simulation conditions and procedure

The simulation followed a factorial design with five manipulated data characteristics including sample size (70, 100, 200, 300, 500, 1000, or 2000), number of classes (2 or 3), number of indicators (4, 5, 6, 7, 8, 9, 10, 11, or 12), quality of indicators (low, moderate, or high), and effect of a covariate on latent class membership (none, small, moderate, or large) (see Table 1 for a summary of the population parameters). The Supplementary Material shows how Mplus 6 (Muthén and Muthén, 1998–2011) was used to generate the data and the Supplementary Material gives an example of how Mplus was used to analyze correctly specified models using maximum likelihood estimation. The true population parameters were used as starting values to decrease computing time and avoid label switching (see discussion below) and local maxima as much as possible (Collins and Lanza, 2010).

**Table 1:** **Summary of population specifications for class proportions, class profiles, and indicator quality (as CRPs)**.

**γ_*c*_**	**Class 1**			**Class 2**			**Class 3**		
	0.67			0.33					
	0.4			0.4			0.2		
	**Quality**			**Quality**			**Quality**		
**Indicator**	**High**	**Moderate**	**Low**	**High**	**Moderate**	**Low**	**High**	**Moderate**	**Low**
1	**0.9**	**0.8**	**0.7**	**0.9**	**0.8**	**0.7**	0.1	0.2	0.3
2	**0.9**	**0.8**	**0.7**	**0.9**	**0.8**	**0.7**	0.1	0.2	0.3
3	**0.9**	**0.8**	**0.7**	0.1	0.2	0.3	0.1	0.2	0.3
4	**0.9**	**0.8**	**0.7**	0.1	0.2	0.3	0.1	0.2	0.3
5	**0.9**	**0.8**	**0.7**	**0.9**	**0.8**	**0.7**	0.1	0.2	0.3
6	**0.9**	**0.8**	**0.7**	0.1	0.2	0.3	0.1	0.2	0.3
7	**0.9**	**0.8**	**0.7**	**0.9**	**0.8**	**0.7**	0.1	0.2	0.3
8	**0.9**	**0.8**	**0.7**	0.1	0.2	0.3	0.1	0.2	0.3
9	**0.9**	**0.8**	**0.7**	**0.9**	**0.8**	**0.7**	0.1	0.2	0.3
10	**0.9**	**0.8**	**0.7**	0.1	0.2	0.3	0.1	0.2	0.3
11	**0.9**	**0.8**	**0.7**	**0.9**	**0.8**	**0.7**	0.1	0.2	0.3
12	**0.9**	**0.8**	**0.7**	0.1	0.2	0.3	0.1	0.2	0.3

All data characteristics were fully crossed, except in one case: for the 3-class LCA models, the 4-indicator condition is generally underidentified (Collins and Lanza, 2010), and so none of these models were studied. There were 7 (sample size) × 9 (number of indicators) × 3 (quality of indicators) × 4 (covariate effect) = 756 2-class conditions, plus 7 × 8 × 3 × 4 = 672 3-class conditions totaling 1428 conditions, with 1000 replications generated for each condition. In cases of non-convergence or potential label switching (see further explanation of exclusion criteria below), we generated additional replications to maintain a balanced design with 1000 replications per cell. However, if more than 50% of a cell's initial replications were unusable due to non-convergence or potential label switching, that individual cell was dropped from the study and not refilled. The simulation conditions were chosen based on previous Monte Carlo studies, common findings in the substantive research, and results from a pilot study.

Following Marsh et al. (1998), we varied the number of indicators between 4 and 12. We studied 2- and 3-class models as these are common in substantive research. In the 2-class models, Class 1 was generated to have a class proportion parameter of γ_1_ = 0.67. Hence, γ_2_ = 0.33 in this condition, because Σγ_*c*_ = 1 by definition in LCA. In the 3-class models, the γ_*c*_ parameters were 0.4, 0.4, and 0.2 for classes 1, 2, and 3, respectively. We chose these class proportions to reflect common class proportions found in substantive research—rarely are all classes exactly equal in size; often at least one class is about twice the size of another class. Class profiles were defined following Collins and Wugalter (1992) and Nylund et al. (2007) such that Class 1 had high CRPs (or class-specific item means) for all items, Class 2 had high CRPs for half of the items, and low for the other half, and in the 3-class models, Class 3 had low CRPs for all items.

All indicators were generated to have two response categories and be locally independent, conditional on latent class membership. Indicators in high quality conditions were generated to either have CRPs (of endorsing the second category) of 0.9 or 0.1, while moderate quality conditions had CRPs of 0.8 and 0.2, and low quality indicators were generated at 0.7 or 0.3, following Collins and Wugalter's (1992) strong and weak measurement strength conditions.

A continuous covariate *X* was generated from a standard normal distribution. The covariate effect sizes were chosen following Rosenthal's (1996) effect size conventions for odds ratios (OR), where *OR* = 1.5, 2.5, and 4 describe small, moderate, and large effect sizes, respectively. The covariate effect was specified in terms of the logistic regression slope coefficients, β_1*c*_ = log(OR), and logistic regression intercept coefficients β_0*c*_. Because the last class serves as reference, only *C*-1 β_1*c*_ parameters were estimated in each model. So in the 3-class models, the β_1*c*_ parameters were specified to be equal within each condition.

When a non-zero covariate effect is included in the model, the intercept parameter β_0*c*_ reflects class proportions when *X* = 0, which in our case was the mean of the covariate. To be consistent with the no-covariate conditions, the covariate intercepts β_0*c*_ were specified to be equal with the same as the unconditional class sizes in the no-covariate conditions.

Even though *N* = 500 has been recommended as a “worthy goal” by Finch and Bronk (2011), a sample of this magnitude may often be unrealistic for applied researchers. Thus, we also studied smaller sample size conditions below *N* = 500. The *N* = 1000 and 2000 conditions were included to explore larger sample sizes in LCA, and whether there is a point at which larger *N* does not continue to meaningfully improve the results.

### Exclusion criteria

We considered four exclusion criteria to determine if a replication was eligible for inclusion in the study: (1) non-convergence, where the estimation failed to provide a complete solution after 500 iterations and a loglikelihood convergence criterion of 0.0000001; (2) label switching, where a complete solution was estimated, but the class labels arbitrarily did not match the class labels of the generated data; (3) incorrigibility, where we were unable to determine whether a replication was correctly labeled; and (4) zero variance of one or more observed variables (this problem only occurred in the 2-class high quality indicator, low sample size conditions, in which 0.2–0.6% of replications per condition had one or more observed variables with zero variance due to sampling error). For each condition, three times the amount of all non-converged, label-switched, incorrigible, and zero-variance replications were re-simulated. We applied the exclusion criteria to these additional replications, and then each cell was refilled with new replications until 1000 proper replications were available for each cell to maintain a balanced design in, so the conditions can be compared using analysis of variance. (Note that while we excluded label switched solutions due to the difficulty they present in aggregating simulation results, such models are unproblematic in practice. However, non-converged or incorrigible solutions would indicate that the model results may not be interpretable). Below we describe in detail how we handled the label-switching issue.

In LCA solutions, there are *c*! possible labeling permutations of *c* classes, so that even with data generated by well-separated and homogeneous classes, it is possible that the parameter estimates may not match the labels of the generated data, which leads to the problem of *label switching*. For applied users of LCA, label switching does not indicate any problem with the estimation *per se*, because class labeling is arbitrary and does not change either the model fit or the interpretation of the classes. Label switching is primarily problematic in the aggregation of results in a simulation study. Although label switching can often be corrected by inspecting the solution, this method can be unreliable and subjective, especially when the estimated parameters vary greatly from the generating parameters (e.g., due to high sampling error in small samples).

In our simulation, we used the true population parameters as starting values for each replication to minimize the occurrence of label switching. In addition, we used an algorithm developed by Tueller et al. (2011) to check whether each replication had correct class labels, incorrect labels, or “incorrigible” labels, meaning the program could not reliably determine if label switching had occurred, due to the class assignment matrix not meeting the algorithm's minimum criteria for class assignment accuracy. Conceptually, incorrigibility indicates that the parameter recovery is so poor (for example, due to high sampling error in small samples) that the population classes cannot be properly identified. Low class assignment accuracy and extremely low parameter recovery is an undesirable quality in LCA models and may suggest that the solution itself is untrustworthy. Thus, we felt that it was acceptable to exclude these replications given that they would be undesirable solutions for substantive researchers. All incorrigible or incorrectly labeled models were counted and excluded from the final analysis.

A trivial amount of replications (0.03%) were detected as label switched and subsequently excluded from further analysis to avoid subjective decisions as to the correct class labels. A more relevant issue was the proportion of incorrigible replications. Although there were very few incorrigible replications in high and moderate indicator quality conditions, some low quality conditions had up to 75% incorrigible replications. In total, 126,653 of the original replications (8.9%) were excluded from the initial analysis due to incorrigibility. Of the 190,896 additional replications that were generated to refill the design, 50,860 replications (26.6%) were excluded for incorrigibility.

**Figure 1:**
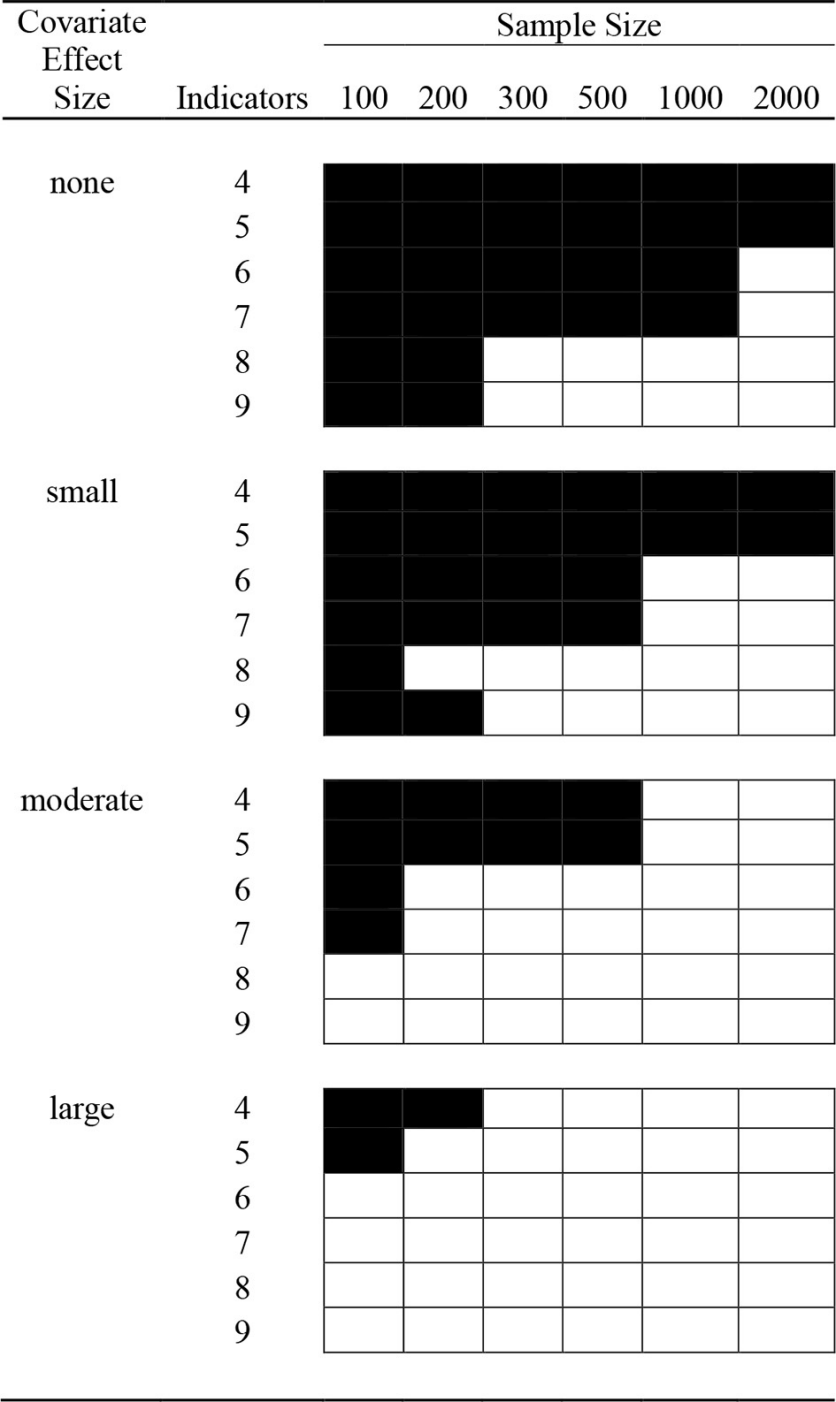
**2-class, low indicator quality excluded conditions**. Blackened cells indicate conditions where at least 50% of the replications were non-converged, label-switched, incorrigible, or had zero-variance.

Preliminary analyses revealed several conditions with a high proportion of replications that met one or more of the four above-mentioned exclusion criteria. We therefore defined a general exclusion criterion for this study at the cell level according to which any condition with less than 50% usable replications was not refilled, but rather excluded entirely from further analysis. We reasoned that conditions with less than 50% usable replications were likely not feasible in practice either due to high levels of non-convergence^1^ or extremely low class assignment accuracy (incorrigibility). All *N* = 70 conditions were excluded according to this criterion, as almost all of these conditions showed high rates of incorrigible or non-converged replications. All other 2-class excluded conditions are shown in Figure 1. It can be seen that in line with our hypotheses, inclusion was positively related to (1) the number of indicators (the more indicators, the more included conditions) and (2) the covariate effect size (the stronger the covariate effect, the more included conditions). The only two 3-class excluded conditions (besides *N* = 70) were *N* = 100 and *N* = 200 with only 5 indicators and a small covariate effect.

### Dependent variables

For the full set of conditions, before exclusion criteria were applied, we examined the number of incorrigible and non-converged replications. Incorrigibility (based on class assignment accuracy) provides a measure of the “messiness” of a solution, that is, the extent to which the original classes in the population are or are not identifiable. For the remaining included cells of the design, we examined the prevalence of boundary parameter estimates and relative parameter estimate bias for class proportions, CRPs, and the covariate regression slopecoefficient.

Relative bias was calculated by subtracting the true value of the parameter from the simulated parameter estimate and dividing the difference by the parameter's true value. In our calculations, we used the absolute value of this bias measure. The absolute value of bias was averaged across all replications within each cell. In the case of CRPs, bias was averaged only among indicators generated with the same population CRP, i.e., in the low-quality condition, the biases of all CRPs generated at 0.7 were averaged, and the biases of all CRPs generated at 0.3 were averaged, so we could determine if bias differed for high vs. low CRPs. Only two “high” CRPs and two “low” CRPs were averaged in each replication to allow for a fair comparison of parameter bias across conditions with different numbers of indicators.

Class proportion estimate bias was calculated separately for each class, given that the classes differed in size. In the covariate conditions, the covariate intercept parameters β_0*c*_ were used to examine bias in the class proportion estimates as they reflect conditional class proportions at *X* = 0, that is, at the mean of the covariate *X*. In the 3-class model, the relative parameter estimate bias of the two β_1*c*_ parameters were averaged, as they were both generated to be the same value.

### Statistical analysis

We examined the impact of sample size, covariate effect size, number of indicators, and quality of indicators, as well as all possible interactions among factors using analysis of variance (ANOVA) for continuous outcomes and logistic regression for binary outcomes (conducted in SPSS 21). After excluding conditions with less than 50% usable replications, 1,160,000 out of 1,428,000 replications (81.2%) were still available for the analysis; such a large sample size has high statistical power to detect even very small effect sizes. In our presentation of the results, we therefore focused on effect size measures, including η^2^ = SS_effect_/SS_total_ for continuous outcomes. Specifically, we focused on factors that showed an effect size of η^2^ > 0.01, independent of other factors in the model. Values of 0.09 > η^2^ ≥ 0.01 are considered “small” effect sizes (Cohen, 1988) and this standard of determining meaningful effects has been used in other simulation studies (Krull and MacKinnon, 1999). We also restricted ourselves to interpreting only 2- and 3-way interactions. For binary outcomes, we used the OR as an effect size, with 2.5 > *OR* ≥ 1.5 considered small effect sizes (Rosenthal, 1996) and worthy of interpretation.

For factors that only surpassed the η^2^ > 0.01 criterion for 2- *or* 3-class conditions, but not both, effect sizes for both conditions were still reported to highlight the differing results between 2- and 3-class conditions. Non-convergence and incorrigibility were examined for all conditions prior to any excluded conditions or refilled replications. Boundary parameter estimates and relative parameter bias were only examined after we applied the exclusion criteria.

## Results

### Incorrigibility

**Figure 2:**
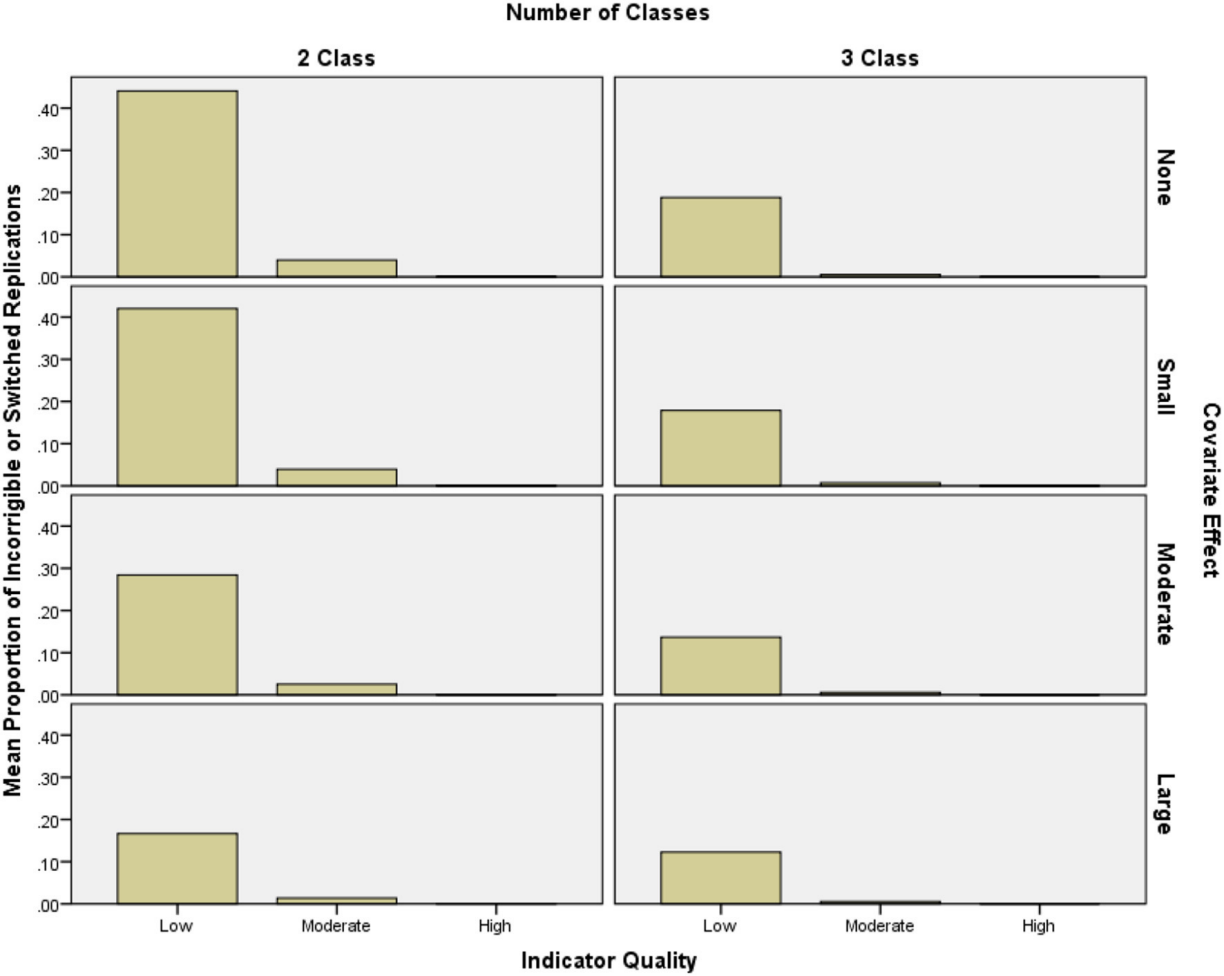
**Mean proportion of incorrigible replications per condition by indicator quality and covariate effect size**.

Incorrigibility ranged between 0 and 74% of replications per condition, and was especially prevalent in conditions with low quality indicators. Improving indicator quality had the highest impact on reducing incorrigibility (2-class *OR* = 11.23, 3-class *OR* = 5.32) while increasing covariate effect size (2-class *OR* = 2.07, 3-class *OR* = 2.49) also had a moderate impact on reducing incorrigibility. The interaction between covariate effect size and quality also had an effect on incorrigibility (2-class *OR* = 1.52, 3-class *OR* = 1.75), such that as indicator quality increased, the impact of the covariate effect size decreased (see Figure 2). Neither sample size (2-class *OR* = 1.11, 3-class *OR* = 1.10), nor the number of indicators (2-class *OR* = 1.08, 3-class *OR* = 1.02), had meaningful effects on incorrigibility.

### Non-converged solutions

**Figure 3:**
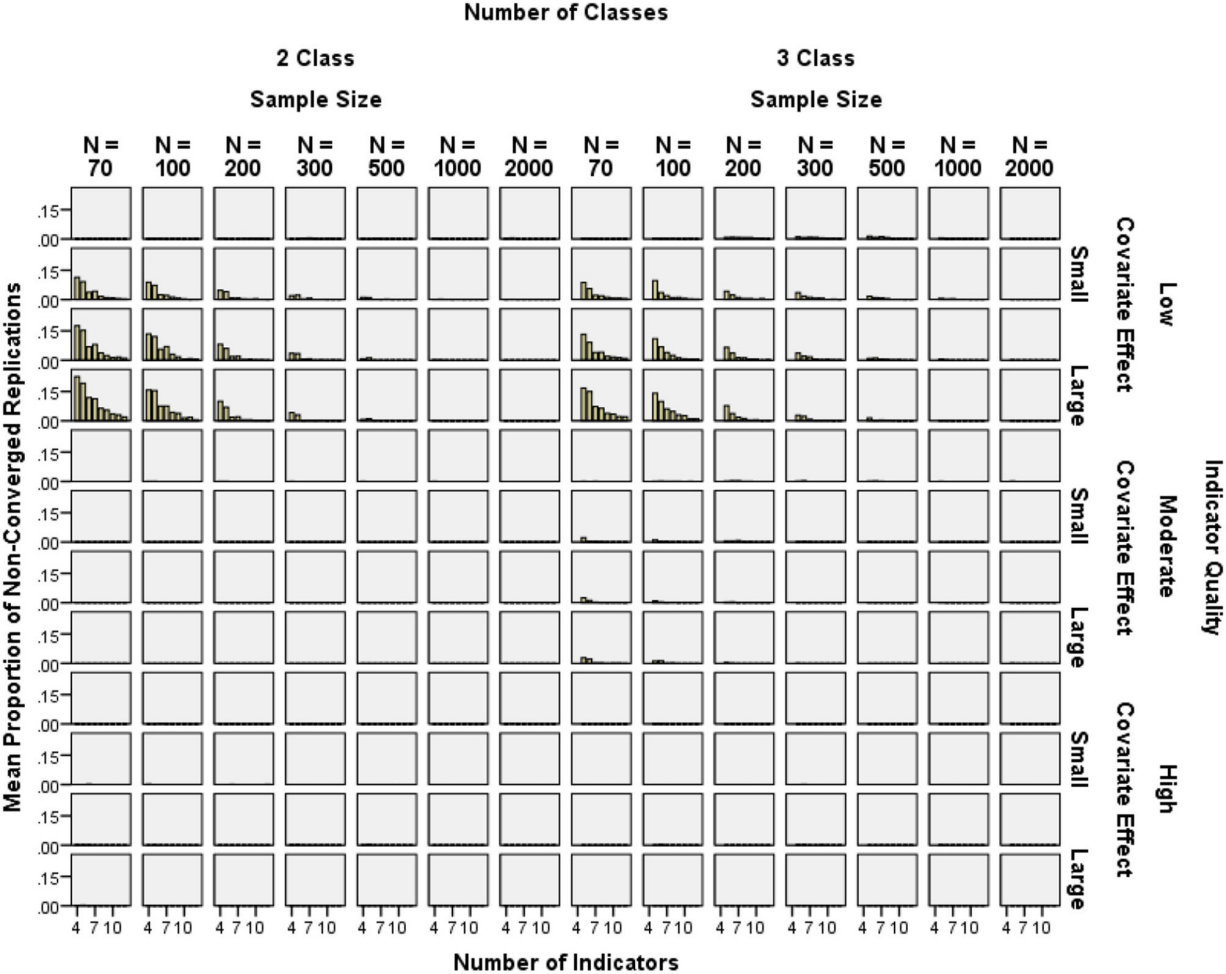
**Mean proportion of non-converged replications by sample size, number of indicators, and covariate effect size for high and low quality indicators**.

In the original set of replications, 0.5% of solutions did not converge; in the refilled set of replications, 1.3% did not converge. Non-convergence was less than 15% in most conditions. Exceptions were *N* = 70 or *N* = 100 with a large or moderate covariate and only 4 or 5 indicators. Figure 3 shows rare non-convergence in high-quality indicator conditions. Higher indicator quality had the largest impact on reducing non-convergence (2-class *OR* = 15.96, 3-class *OR* = 4.34), while decreasing covariate effect size (2-class *OR* = 3.25, 3-class *OR* = 2.10), increasing the number of indicators (2-class *OR* = 1.78, 3-class *OR* = 1.74), and increasing the sample size (2-class *OR* = 1.50, 3-class *OR* = 1.33) also reduced non-convergence (see Figure 3).

### Boundary parameter estimates

**Figure 4:**
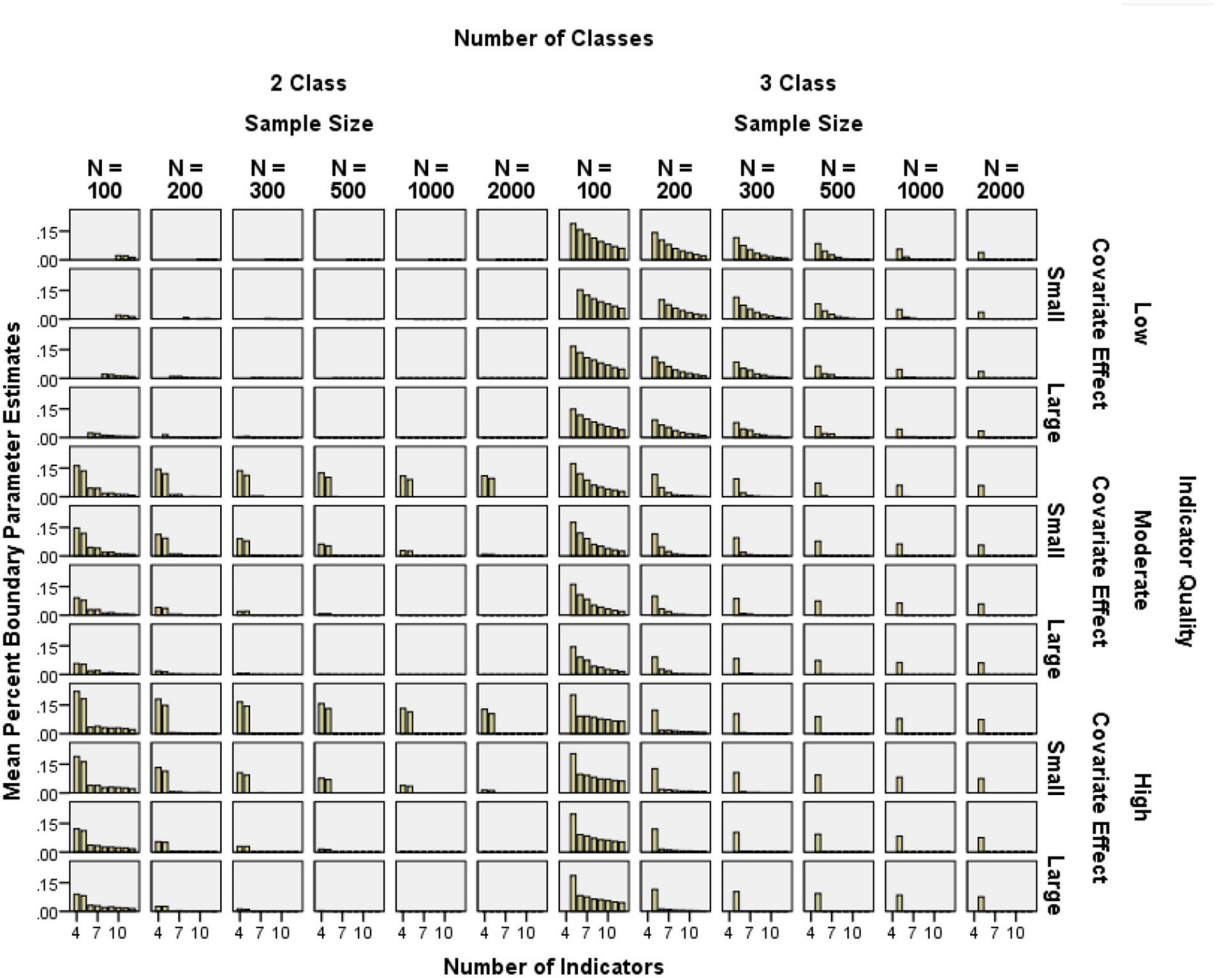
**Mean percent of boundary parameter estimates by sample size, number of indicators, indicator quality, and covariate effect size**.

**Figure 5:**
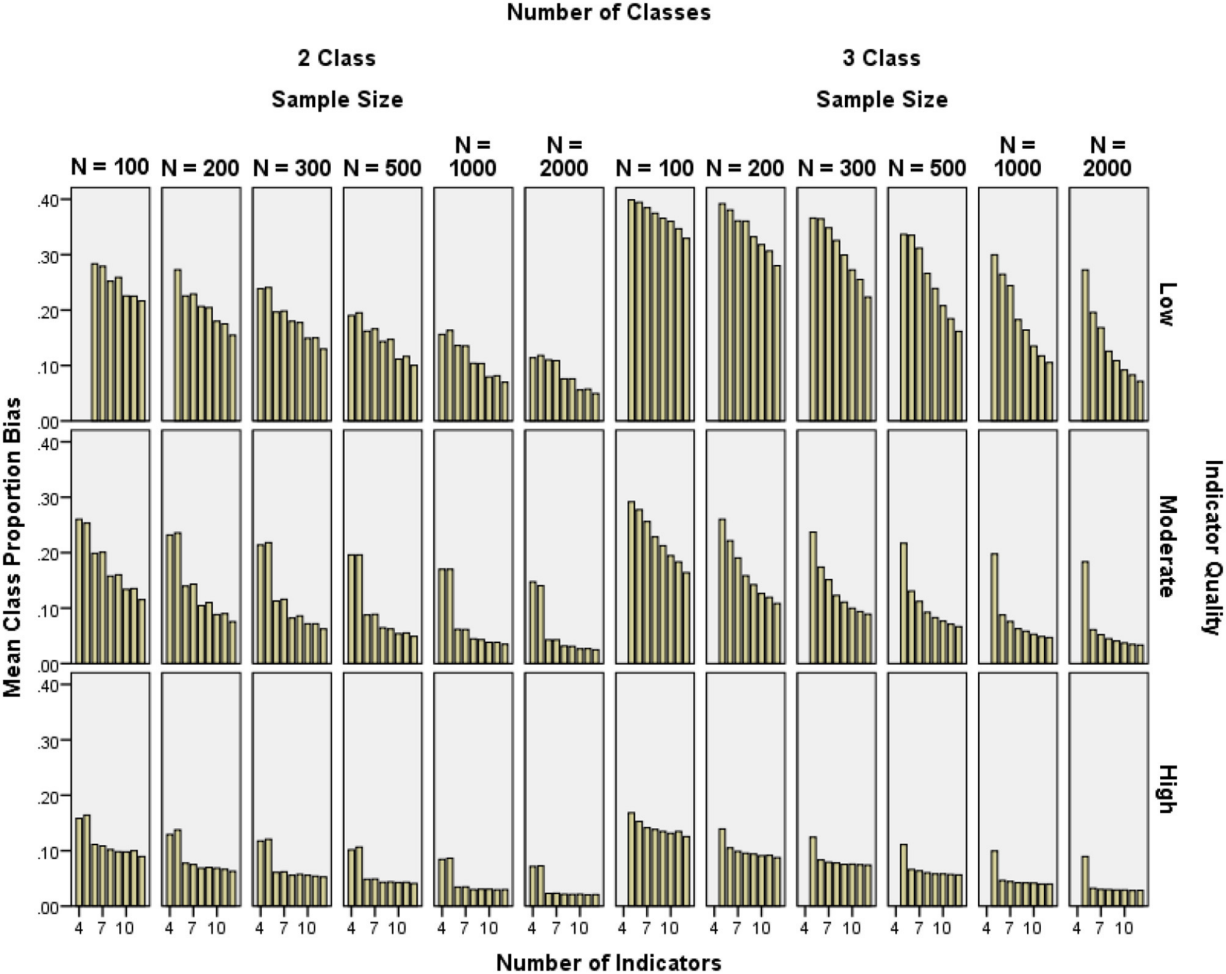
**Mean class proportion relative bias by indicator quality, number of indicators, and sample size**.

**Figure 6:**
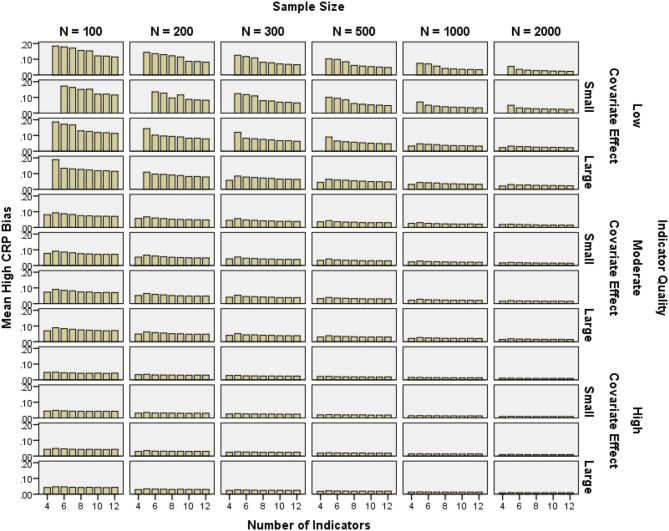
**Mean high conditional response probability relative bias by number of indicators, indicator quality, covariate effect size, and sample size**.

**Figure 7:**
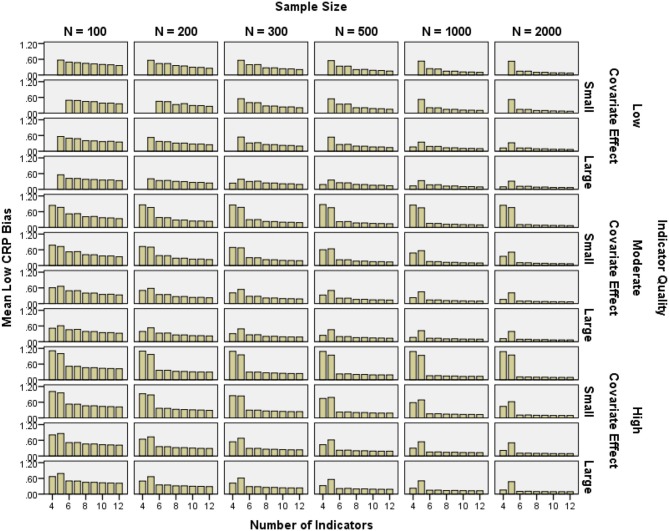
**Mean low conditional response probability relative bias by number of indicators, indicator quality, covariate effect size, and sample size**.

**Figure 8:**
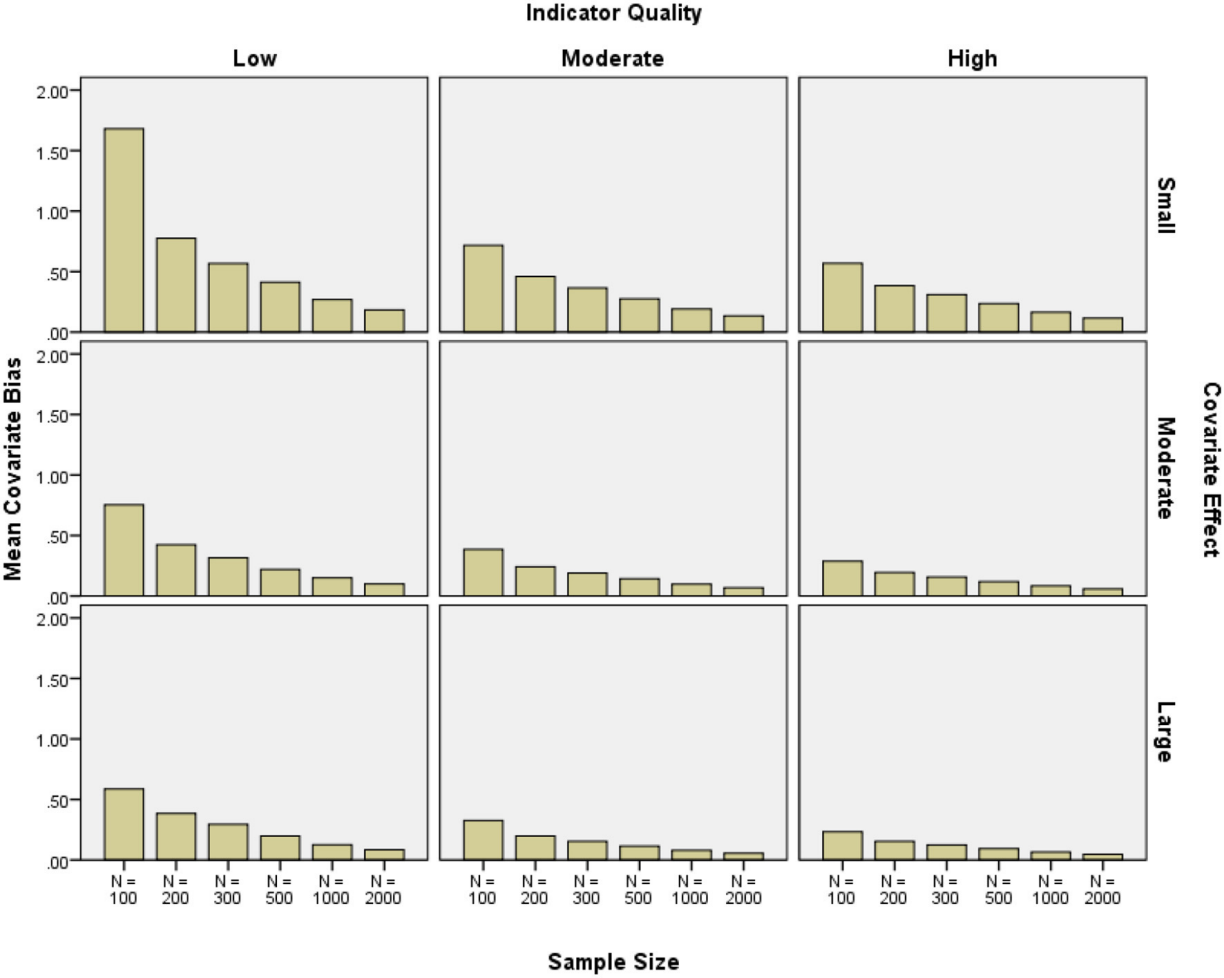
**Mean covariate relative bias by indicator quality, covariate effect size, and sample size**.

The frequency of boundary parameter estimates was assessed by calculating the proportion of boundary parameter estimates per total number of independent CRP parameters in each condition. Boundary parameter prevalence was generally lower than 15%, except with *N* = 100 and five or fewer indicators (see Figure 4). Note that in Figures 4–**8**, empty cells in low indicator quality conditions correspond to conditions which had more than 50% unusable replications, as explained in the exclusion criteria above.

Boundary parameter prevalence was reduced by decreasing the indicator quality (2-class η^2^ = 0.079, 3-class η^2^ = 0.011), increasing the number of indicators (2-class η^2^ = 0.039, 3-class η^2^ = 0.218), increasing the covariate effect size (2-class η^2^ = 0.010, 3-class η^2^ = 0.003), and increasing the sample size (2-class η^2^ = 0.001, 3-class η^2^ = 0.211).

Covariate effect size and indicator quality interacted (2-class η^2^ = 0.021, 3-class η^2^ = 0.001) such that stronger covariates helped decrease the “negative” impact of high quality indicators. Covariate effect size and the number of indicators interacted (2-class η^2^ = 0.048, 3-class η^2^ = 0.001) such that the impact of increasing covariate effect size decreased with more indicators. Indicator quality and number of indicators interacted (2-class η^2^ = 0.078, 3-class η^2^ = 0.012), such that as number of indicators increased, the negative impact of the high indicator quality decreased. Similarly, as sample size increased, the negative impact of the high indicator quality decreased (2-class η^2^ = 0.001, 3-class η^2^ = 0.013), and as the number of indicators increased, the effect of sample size decreased (2-class η^2^ = 0.005, 3-class η^2^ = 0.026).

Also, in the 2-class conditions, all 3-way interactions showed η^2^ ≥ 0.01. These 3-way interactions were such that increasing one factor reduced the impact of other 2-way interactions. For example, increasing the number of indicators attenuated the interaction between quality and covariate effect size (2-class η^2^ = 0.096). Similarly, increasing the sample size attenuated the interactions between quality and covariate effect size (2-class η^2^ = 0.018), covariate effect size and number of indicators (2-class η^2^ = 0.016), and quality and number of indicators (2-class η^2^ = 0.010). These effects did not appear in the 3-class conditions.

With few indicators (4 or 5), the high quality conditions had more boundary parameter estimates than the low quality conditions. However, except for the 4- and 5-indicator conditions, prevalence of boundary estimates was very similar among low and high quality conditions. In summary, using high quality indicators seemed only problematic in small samples and/or with few indicators.

### Parameter bias

#### Class proportion bias

Class proportion bias was generally below 10% for moderate and high indicator quality conditions with at least 6 indicators and *N* ≥ 300, but was high in low indicator quality conditions (see Figure 5). Class 1 proportion bias was reduced by increasing indicator quality (2-class η^2^ ≥ 0.052, 3-class η^2^ = 0.109), increasing the number of indicators (2-class η^2^ = 0.044, 3-class η^2^ = 0.034), and increasing the sample size (2-class η^2^ = 0.056, 3-class η^2^ = 0.046). Similar effect sizes were found for Class 2 and Class 3 bias.

#### CRP bias

CRP bias was calculated including boundary parameter estimates, because in practice, researchers would also typically interpret boundary estimates for an otherwise proper solution. It was important to know whether including boundary parameters led to bias. In addition, in some replications with few indicators, all low probability CRPs (0.1, 0.2, or 0.3) were estimated at zero and so bias could not have been calculated without boundary parameters.

CRP bias for high probability indicators was below 10% in all high quality and most moderate quality conditions, as well as low quality conditions with *N* ≥ 500 (see Figure 6). Low probability CRP bias was generally below 10% with *N* = 2000 and 6–12 indicators. Low probability CRP bias in the 4- and 5-indicator conditions was often near 100% (see Figure 7). Note that relative bias was calculated as the absolute value of the difference between the mean parameter estimate and the population value of the parameter, then divided by the population value. Even if the raw difference is the same for two parameters, the *relative* bias for the parameter with a smaller population value would be higher. The higher relative bias for low CRPs and for smaller class proportions was due at least in part to this mathematical property, which made the results in Figure 7 look more extreme.

High probability CRP bias was reduced by increasing indicator quality (2-class η^2^ = 0.049, 3-class η^2^ = 0.111), as well as sample size (2-class η^2^ = 0.116, 3-class η^2^ = 0.113). Indicator quality and sample size interacted such that the impact of greater sample size decreased with increasing indicator quality (2-class η^2^ = 0.012, 3-class η^2^ = 0.025). Low probability CRP bias was partly reduced by increasing covariate effect size (2-class η^2^ = 0.019, 3-class η^2^ = 0.000), the number of indicators (2-class η^2^ = 0.081, 3-class η^2^ = 0.139), and sample size (2-class η^2^ = 0.061, 3-class η^2^ = 0.069). Indicator quality and the number of indicators interacted such that the impact of more indicators decreased with increasing indicator quality (2-class η^2^ = 0.003, 3-class η^2^ = 0.011). Also, covariate effect size and the number of indicators interacted such that the impact of greater sample size decreased with increasing covariate effect size (2-class η^2^ = 0.003, 3-class η^2^ = 0.011).

#### Covariate bias

Covariate parameter estimate bias was large when the covariate effect, indicator quality, and sample size were small. The same type of bias was generally less than 10% with high or moderate quality indicators and a moderate or large covariate effect with *N* = 1000 to *N* = 2000 (see Figure 8). Covariate bias was reduced by increasing the sample size (2-class η^2^ = 0.096, 3-class η^2^ = 0.001), as well as the covariate effect size (2-class η^2^ = 0.029, 3-class η^2^ = 0.001), and indicator quality (2-class η^2^ = 0.022, 3-class η^2^ = 0.000). Three-class conditions showed no factors with η^2^ > 0.01 overall. The number of indicators did not have a meaningful effect on covariate bias (2-class η^2^ = 0.007, 3-class η^2^ = 0.000).

## Discussion

With LCA becoming increasingly popular across diverse fields within the social sciences, it is important for researchers to know which factors influence the quality of estimation when using this method. Although past research suggests a minimum sample size of *N* = 500 for LCA (Finch and Bronk, 2011), as well as the benefits of having more indicators (Collins and Wugalter, 1992; Marsh et al., 1998; von Oertzen et al., 2010), higher quality indicators (Collins and Wugalter, 1992), and including a covariate of class membership, these findings have either been taken from the SEM or general mixture modeling literature, or they are not based on comprehensive simulation work. To our knowledge, this study is the first to study more systematically these factors and their interplay in LCA under a large set of conditions. Below, we summarize our main findings and explain which factors improve LCA performance.

### Sample size

Many applied researchers face limitations in terms of the size of the samples that they can gather, so it is important to understand which factors can be beneficial when a sample size of 500 as recommended by Finch and Bronk (2011) may simply not be available. We found support for the hypothesis that using more and high quality indicators or a covariate that is strongly related to class membership can alleviate some of the problems frequently found with small sample sizes. Nonetheless, there was a relatively clear limit for the minimum sample size. We found, for example, that conditions of *N* = 70 were not feasible under virtually any condition we examined: either there were too many convergence problems, or the class assignment accuracy was too low to clearly interpret the classes with this sample size.

Sample size itself showed a small impact in decreasing non-convergence, and a moderate impact on decreasing boundary parameter estimates, as well as class proportion, low CRP, and covariate effect bias. Sample size interacted with indicator quality such that as sample size increased, the negative impact of CRPs of 0.1 or 0.9 on the number of boundary parameters decreased. Sample size also interacted with the number of indicators in reducing boundary parameter estimates such that using many indicators could compensate for a small sample size. Sample size further interacted with indicator quality in reducing low CRP and Class 3 proportion bias such that high indicator quality could compensate for low sample size. These findings highlight what factors can compensate for a lower sample size—higher number and quality of indicators, adding a strong covariate—and which conditions require higher sample sizes—lower number and quality of indicators.

### Number of indicators

One of the key factors examined here was the influence of the number of indicators, and whether adding more indicators to an LCA is beneficial rather than detrimental. In line with the results of Marsh et al. (1998) for SEM with continuous variables, and Collins and Wugalter (1992) for LTA, we found that using more indicators in LCA is generally beneficial. Increasing the number of indicators had a large effect on decreasing the occurrence of solutions with low class assignment accuracy. This makes sense, given that more indicators contribute to greater certainty in defining classes. Using more indicators also improved convergence rates and led to reduced class proportion and low probability CRP bias. Also, the number of indicators interacted with indicator quality such that using more indicators negated the negative impact of using indicators with CRPs close to zero or one on boundary parameter estimates. Furthermore, the number of indicators interacted with covariate effect size in reducing low CRP bias such that using more indicators could partly compensate for a small covariate effect size.

Our results demonstrate that, at least under conditions similar to the ones studied here, researchers have no reason to avoid adding more indicators in an attempt to prevent data sparseness^2^. In fact, we found that adding more indicators *decreased* the likelihood of boundary parameter estimates, which often arise from data sparseness.

Note that the conditions with the lowest number of indicators (i.e., the 4- and 5-indicator models) were very problematic, with most frequent boundary parameter estimates and often the highest parameter bias. Many of the low quality 4- and 5-indicator conditions were ultimately excluded from the analysis because of high levels of non-convergence and incorrigibility. This could be because the particular population class profiles chosen for these models resulted in a larger number of empirically underidentified solutions. Replications may have passed the Mplus criterion for (under)identification while in fact, they were close to empirically underidentified. Further research should examine whether 4- and 5-indicator models are generally problematic, or whether they perform better with different class profiles. In addition, further studies should examine whether there is a point at which adding more indicators causes problems. Based on our findings, we recommend avoiding designs with fewer than 5 indicators.

### Indicator quality

We also examined whether higher quality indicators are always better, and if indicator quality can compensate for a small sample size. The answer here is for the most part yes—increasing indicator quality almost always improved outcomes, even beyond just parameter recovery (Collins and Wugalter, 1992).

Higher indicator quality had a small to moderate effect on decreasing incorrigibility and a large effect on improving convergence rates. Improving indicator quality also had a small effect on decreasing class proportion, covariate, and high CRP bias. Furthermore, the impact of indicator quality interacted with the impact of adding a covariate on decreasing incorrigibility, such that higher quality indicators could partly compensate for a low covariate effect size. Indicator quality also interacted with the number of indicators such that higher quality indicators could compensate for a low number of indicators in reducing low CRP bias.

The only outcome for which high quality indicators performed poorly was the prevalence of boundary parameter estimates, most likely because CRPs of 0.9 or 0.1 are very close to one or zero and so are most easily estimated at the boundary, especially in smaller samples due to increased sampling error (Galindo-Garre and Vermunt, 2006). However, boundary parameter estimates did occur in low and moderate indicator quality conditions as well, suggesting that high quality indicators are not the only factor that causes boundary estimates. Furthermore, the “negative impact” of high quality indicators on boundary estimates decreased as the number of indicators increased. As discussed previously, this may indicate that using too few indicators of any quality may result in unstable estimation and frequent boundary parameter estimates.

This discussion, of course, is predicated on the idea that boundary parameter estimates are inherently problematic. Although they still may present interpretational difficulties, there were many conditions with high boundary parameter prevalence that did not show any other negative outcomes. Moreover, we included boundary parameter estimates in our calculation of CRP bias and found that in many conditions, the bias was still acceptably low. This suggests that boundary parameter estimates may not be problematic in general, although further research on this matter is clearly needed. Taken together, our results suggest that higher quality indicators should be used whenever possible, with the understanding that boundary estimates may be more likely to occur if the number of indicators is small to modest.

### Use of a covariate

Our results indicate that adding a covariate with a larger effect size to predict class membership in the model may also be beneficial to LCA model estimation in general. We found covariates to have a moderate impact on decreasing incorrigibility. The covariate effect size factor had a small effect on decreasing boundary parameter estimates and low CRP and covariate bias. Covariate effect size also interacted with indicator quality in decreasing incorrigibility such that a large covariate effect size could to some extent compensate for using low-quality indicators. Covariate effect size also interacted with the number of indicators such that having a larger covariate effect size could compensate for using few indicators in reducing low CRP bias.

This extends upon previous findings according to which it is beneficial to add a covariate to a mixture model (Muthén, 2004; Vermunt and Magidson, 2005). In particular, the moderate effect of covariate size on decreasing number of incorrigible replications follows the findings by Lubke and Muthén (2007) who showed that adding a covariate increases proper class assignment. These findings provide further support for the recommendation that including significant covariates of class membership in an LCA model is generally beneficial and can in fact offset other suboptimal conditions, because the covariate provides additional information that can be used in the estimation process.

Note, however, that a larger covariate effect size had a moderate effect on increasing non-convergence in our study. This mainly occurred in small sample size conditions (*N* = 70 to *N* = 200) with few (4–5) indicators. Still, these conditions were problematic even without a covariate—perhaps adding a covariate to an already unstable model created an untenable estimation burden that caused the model to fail. Even then, with at least *N* = 100, non-convergence rates rarely surpassed 15% in general.

Although we found the inclusion of covariates to be beneficial for the LCA estimation in general, the logistic regression (β_1*c*_) coefficients that reflect the covariate effects themselves showed relatively high biases unless the sample sizes were very large and covariate effects were at least moderate in size. A more detailed analysis of the Monte Carlo sampling distributions of the β_1*c*_ coefficients revealed that these parameters had a larger variability and more outliers than the other types of LCA parameters examined here. This shows that the point estimates of the β_1*c*_ coefficients need to be interpreted somewhat cautiously in practice, at least in small samples and when covariate effects are only small or moderate in size.

### Planning an LCA study

Our results provide evidence that the use of a larger number of high-quality indicators and the inclusion of at least one strong covariate positively affect LCA model estimation, and that these factors can sometimes compensate for other suboptimal conditions (e.g., a relatively small sample size). When planning an LCA study, researchers can draw upon these findings to use LCA most efficiently. Note that we are not suggesting that researchers should add indicators or covariates to an LCA without a theoretical basis. Instead, we have found that researchers often wish to conduct LCA with a set of theoretically relevant variables, but do not know if using a partial or full set of variables is more desirable. Researchers often worry that using their full set of indicators with a small sample size may lead to data sparseness and other estimation problems. These concerns may also apply to those researchers who are interested in a covariate's effect on latent class membership and who may be concerned that the extra parameters will negatively affect outcomes, such as parameter bias and convergence. Under the conditions examined here, we suggest that researchers can in general feel comfortable using a larger set of indicators and adding theoretically meaningful covariates to the model.

Not surprisingly, using a larger sample size will usually result in better model estimation. However, large sample sizes are not always available. Instead, the careful choice of (a possibly large number of) high quality indicators and strong covariates of class membership can—in part—offset the detrimental effects of a limited sample size, although caution needs to be applied in interpreting covariate effects with smaller sample sizes. The acceptable minimum *N* depends of course on the specific conditions in the application at hand—rarely will actual data characteristics exactly match the simulation conditions examined here.

On the other hand, the combination of small sample size and low-quality indicators resulted in a high proportion of non-converged or incorrigible solutions in our simulation. Even though it may be possible to estimate an LCA model with *N* < 100, our simulation study suggests that such solutions should be interpreted with great caution, given the high prevalence of “messy” solutions. Given that we correctly specified all models and provided good starting values, non-convergence in these conditions is probably due to empirical underidentification, which generally includes highly unstable parameter estimates. Incorrigibility was assessed based on class assignment accuracy: poor class assignment accuracy is also associated with problems like non-convergence or non-positive definite latent variable matrices (Tueller et al., 2011). Conditions with high proportions of models that did not converge or had poor class assignment accuracy suggest that parameter estimates in these conditions are likely unstable. Unstable parameters are difficult to reproduce in a different sample, so results based on these unstable parameters should not be generalized to a larger population or other subsample.

Although our findings provide a good starting point, we recommend that researchers who are uncertain about the minimum feasible sample size conduct their own application-oriented simulation study as described in Muthén and Muthén (2002). In addition, it may be useful for researchers to conduct pilot studies to identify the best latent class indicators and covariates from a larger set of variables before conducting the actual study.

### Limitations and suggestions for future research

There are ways in which every simulation study is limited. In particular, the results of simulation studies may not generalize to conditions beyond the ones examined in the study. The simulated data were specified so that within each model, there were only two values of CRPs. Also, the number of classes (2 or 3) and relative class proportions were confounded with the class profiles. The results of this study may not apply to models with more classes, much smaller classes, or models with classes that are equal in size.

Similarly, only 2- and 3-class models were studied here, and the results, particularly with regard to sample size, likely do not generalize to models with more classes. As each additional class is added to a model, the number of parameters estimated as well as the number of cells in the data contingency table (and thus data sparseness) increases. For models with more classes, there may be a minimum sample size for which adding more indicators is beneficial, and this should be studied in future research. Furthermore, our method of refilling cells with converged, corrigible replications may have resulted in overly optimistic estimations of parameter bias. Different bias may have been seen if the bias could have been calculated from the excluded replications as well. Nonetheless, refilling cells to maintain a balanced design is common practice in simulation research. In addition, additional bias may have resulted from analyzing only the subset of initially completed replications, so this problem cannot be easily resolved.

Although a wide variety of conditions were examined here, there still remain many other factors. In particular, in practice researchers rarely use a set of indicators with equal CRPs as done in the present study. Future research should consider mixed indicator quality conditions. Also, we only studied binary indicators. Future research should examine whether the results hold for polytomous and continuous indicators as well. A preliminary simulation conducted by our group with continuous indicators (i.e., latent profile analysis) showed similar findings regarding the beneficial aspects of using many high quality indicators and a strong covariate. Furthermore, we only studied models with a single covariate. Future research should study the effects of including multiple (and potentially correlated) covariates in LCA.

## Author note

Ingrid C. Wurpts, Department of Psychology, Arizona State University. Christian Geiser, Department of Psychology, Utah State University. Some of the results presented in this article are based on Ingrid Wurpts' Master's thesis. An earlier version of this article was presented at the Modern Modeling Methods conference in Storrs, Connecticut, May 22–23, 2012. We are grateful to Leona Aiken and Stephen West for their helpful comments on the draft.

### Conflict of interest statement

The authors declare that the research was conducted in the absence of any commercial or financial relationships that could be construed as a potential conflict of interest.

## Footnotes

^1^ Note that we used the Mplus 6 default convergence criteria, and all models were correctly specified and given starting values based on the true population parameters. If a replication did not converge under these conditions, this was a good indication that there was a problem with the model (such as empirical underidentification or a local maximum solution), rather than just a problem of using too few iterations or bad starting values.

^2^Note that increased data sparseness due to a larger number of indicators may still cause issues with model fit assessment (e.g., Langeheine et al., 1996) that were not studied here.

## References

[B2] Clark S. Muthén B. (2009). Relating Latent Class Analysis Results to Variables not Included in the Analysis. Available online at: http://www.statmodel.com/download/relatinglca.pdf

[B4] Cohen J. (1988). Statistical Power Analysis for the Behavioral Sciences. Hillsdale, NJ: Lawrence Erlbaum

[B5] Collins L. M. Lanza S. T. (2010). Latent Class and Latent Transition Analysis: With Applications in the Social, Behavioral, and Health Sciences. Hoboken, NJ: John Wiley & Sons Inc

[B6] Collins L. M. Wugalter S. E. (1992). Latent class models for stage-sequential dynamic latent variables. Multivariate Behav. Res. 27, 131–157 10.1207/s15327906mbr2701_8

[B7] Crow S. J. Swanson S. A. Peterson C. B. Crosby R. D. Wonderlich S. A. Mitchell J. E. (2011). Latent class analysis of eating disorders: relationship to mortality. J. Abnorm. Psychol. 121, 225–231 10.1037/a0024455 21707126 PMC8667201

[B8] Finch W. H. Bronk K. C. (2011). Conducting confirmatory latent class analysis using Mplus. Struct. Equ. Modeling 18, 132–151 10.1080/10705511.2011.532732

[B9] Galindo-Garre F. Vermunt J. (2006). Avoiding boundary estimates in latent class analysis by Bayesian posterior mode estimation. Behaviormetrika 33, 43–59 10.2333/bhmk.33.43

[B10] Geiser C. Lehmann W. Eid M. (2006). Separating “rotators” from “non-rotators” in the mental rotations test: a multigroup latent class analysis. Multivariate Behav. Res. 41, 261–293 10.1207/s15327906mbr4103_2 26750337

[B33] Krull J. L. MacKinnon D. P. (1999). Multilevel mediation modeling in group-based intervention studies. Eval. Rev. 23, 418–444 10.1177/0193841X9902300404 10558394

[B13] Langeheine R. Pannekoek J. Van De Pol F. (1996). Bootstrapping goodness-of-fit measures in categorical data analysis. Sociol. Methods Res. 24, 492–516 10.1177/0049124196024004004

[B15] Lubke G. Muthén B. O. (2007). Performance of factor mixture models as a function of model size, covariate effects, and class-specific parameters. Struct. Equ. Modeling 14, 26–47 10.1207/s15328007sem1401_2

[B16] Marsh H. Hau K. Balla J. Grayson D. (1998). Is more ever too much? The number of indicators per factor in confirmatory factory analysis. Multivariate Behav. Res. 33, 181–220 10.1207/s15327906mbr3302_1 26771883

[B18] Muthén B. O. (2004). Latent variable analysis: growth mixture modeling and related techniques for longitudinal data, in Handbook of Quantitative Methodology for the Social Sciences, ed Kaplan D. (Newbury Park, CA: Sage), 345–368 10.4135/9781412986311.n19

[B19] Muthén L. K. Muthén B. O. (1998–2011). Mplus User's Guide, 6th Edn Los Angeles, CA: Muthén & Muthén

[B20] Muthén L. K. Muthén B. O. (2002). How to use a Monte Carlo study to decide on sample size and determine power. Struct. Equ. Modeling 9, 599–620 10.1207/S15328007SEM0904_8

[B21] Nylund K. L. Asparouhov T. Muthén B. O. (2007). Deciding on the number of classes in latent class analysis and growth mixture modeling: a Monte Carlo simulation study. Struct. Equ. Modeling 14, 535–569 10.1080/10705510701575396

[B22] Peugh J. Fan X. (2013). Modeling unobserved heterogeneity using latent profile analysis: a Monte Carlo simulation. Struct. Equ. Modeling 20, 616–639 10.1080/10705511.2013.824780

[B23] Rosenthal J. A. (1996). Qualitative descriptors of strength of association and effect size. J. Soc. Serv. Res. 21, 37–59 10.1300/J079v21n04_02

[B24] Steinley D. Brusco M. J. (2011). Evaluating mixture modeling for clustering: recommendations and cautions. Psychol. Methods 16, 63 10.1037/a0022673 21319900

[B25] Tein J. Y. Coxe S. Cham H. (2013). Statistical power to detect the correct number of classes in latent profile analysis. Struct. Equ. Modeling 20, 640–657 10.1080/10705511.2013.824781 24489457 PMC3904803

[B26] Tueller S. J. Drotar S. Lubke G. H. (2011). Addressing the problem of switched class labels in latent variable mixture model simulation studies. Struct. Equ. Modeling 18, 110–131 10.1080/10705511.2011.534695

[B27] Tueller S. J. Lubke G. H. (2011). Evaluation of structural equation mixture models parmeter estimates and correct class assignment. Struct. Equ. Modeling 17, 165–192 10.1080/10705511003659318 20582328 PMC2890304

[B28] Uebersax J. (2000). A Brief Study of Local Maximum Solutions in Latent Class Analysis. Available online at: http://www.john-uebersax.com/stat/local.htm

[B29] Vermunt J. K. (2003). Multilevel latent class models. Sociol. Methodol. 33, 213–239 10.1111/j.0081-1750.2003.t01-1-00131.x

[B31] Vermunt J. K. Magidson J. (2004). Latent class analysis, in The Sage Encyclopedia of Social Sciences Research Methods, eds Lewis-Beck M. S. Bryman A. Liao T. F. (Thousand Oakes, CA: Sage Publications), 549–553

[B32] Vermunt J. K. Magidson J. (2005). Structural equation models: mixture models, in Encyclopedia of Statistics in Behavioral Science, eds Everitt B. Howell D. (Chichester: Wiley), 1922–1927 10.1002/0470013192.bsa600

[B34] von Oertzen T. Hertzog C. Lindenberger U. Ghisletta P. (2010). The effect of multiple indicators on the power to detect inter-individual differences in change. Br. J. Math. Stat. Psychol. 63, 627–646 10.1348/000711010X486633 20211053

