# Supplementary material for: Is adding more indicators to a latent class analysis beneficial or detrimental? Results of a Monte-Carlo study
Source: Front Psychol. 2014 Aug 21;5:920. doi: 10.3389/fpsyg.2014.00920 (PMC4140387; doi:10.3389/fpsyg.2014.00920)
Supplement: Supplementary file 1 [file DataSheet1.DOCX]

APPENDIX A

Mplus syntax to generate data: data must be generated and analyzed with separate syntax files so that parameter estimates and class assignment probabilities from each replication can be saved individually. These results and class assignment probabilities are then input into the label-switching R program as described in Tueller, Drotar, and Lubke (2011).

TITLE: Monte Carlo generate LCA covariate effect = large classes = 2

indicators = c1-c4 quality = 1 estimator = ml sample size = 70

MONTECARLO:

NAMES = c1-c4 x; !Define variable names;

NOBSERVATIONS = 70; !Set sample size;

NREPS = 1000; !Set number of replications;

SEED = 46523; !Set seed for random number generator;

GENCLASSES = L(2); ! Assign name “L” to the categorical latent

variable in generation model, and specify latent variable L has two categories;

CLASSES = L(2); ! Assign name “L” to the categorical latent variables

in the analysis model and specify latent variable L has two

categories;

REPSAVE = ALL; !Save generated data for each replication separately;

SAVE = 1211111_*.dat; !Choose file name for generated data;

GENERATE = c1-c4 (1); !Define latent class indicators to have one

threshold, or be binary, in generation model;

CATEGORICAL = c1-c4; !Define latent class indicators as binary in

estimation model;

MODEL POPULATION: !Define data generation model;

%OVERALL% !Define parameters that apply to both classes;

[x@0]; !Generate covariate X with mean of zero;

x@1; !Generate covariate X with variance of one;

L#1 on x*1.38629; !Specify logistic regression slope coefficient for regression of classes on covariate X;

[L#1*.7082]; !Specify logistic regression intercept coefficient;

%L#1% !Define parameters that apply to latent class 1;

[c1$1*-2.197]; !Specify logit threshold for class 1 indicators;

[c2$1*-2.197];

[c3$1*-2.197];

[c4$1*-2.197];

%L#2% !Define parameters that apply to latent class 2;

[c1$1*-2.197]; !Specify logit threshold for class 2 indicators;

[c2$1*-2.197];

[c3$1*2.197];

[c4$1*2.197];

ANALYSIS: type = mixture; !Estimate a mixture model;

starts = 0; !Turn off random starts;

UCELLSIZE = 0; !Avoid deleting any cells in computation of chi-square;

ESTIMATOR = ml; !Specify maximum likelihood estimation;

PROCESS = 8; !Use 8 processors;

MODEL: !Define data analysis model with starting values based on data generation parameters;

%OVERALL%

L#1 on x*1.38629;

[L#1*.7082];

%L#1%

[c1$1*-2.197];

[c2$1*-2.197];

[c3$1*-2.197];

[c4$1*-2.197];

%L#2%

[c1$1*-2.197];

[c2$1*-2.197];

[c3$1*2.197];

[c4$1*2.197];

APPENDIX B

Mplus syntax to estimate correctly specified LCA model on simulated data

TITLE: Monte Carlo estimate LCA covariate effect = large classes = 2

indicators = c1-c4 quality = 1 estimator = ml sample size = 70

DATA:

FILE = 1211111_1.dat; !Specify data filename;

VARIABLE:

NAMES = c1-c4 x tc; !List latent class indicators, covariate, and ID

variable;

CATEGORICAL = c1-c4; !Specify latent class indicators as binary;

CLASSES = L(2); !Specify one latent categorical variable L, with two

classes;

IDVAR IS tc; !Give each observation and ID and name the ID variable

“tc.” This is necessary to use the label switch detection program;

ANALYSIS: type = mixture; !Estimate a mixture model;

starts = 0; !Turn off random starts;

UCELLSIZE = 0; !Avoid deleting any cells in computation of chi-square;

ESTIMATOR = ml; !Specify maximum likelihood estimation;

PROCESSORS = 8; !Use 8 processors;

MODEL: !Define data analysis model with starting values based on data generation parameters;

%OVERALL%

L#1 on x*1.38629 (b01); !Specify logistic regression slope coefficient for regression of classes on covariate X;

[L#1*.7082](L1); !Specify logistic regression intercept coefficient;

%L#1%

[c1$1*-2.197](c1_1); !Estimate latent class indicator logit thresholds

for class 1 and call them “c1_1”, etc;

[c2$1*-2.197](c2_1);

[c3$1*-2.197](c3_1);

[c4$1*-2.197](c4_1);

%L#2%

[c1$1*-2.197](c1_2); !Estimate latent class indicator logit thresholds

for class 2 and call them “c1_2”, etc;

[c2$1*-2.197](c2_2);

[c3$1*2.197](c3_2);

[c4$1*2.197](c4_2);

MODEL CONSTRAINT: !Use MODEL CONSTRAINT to covert covariate effect into odds ratio scale and convert logit thresholds into probability scale;

NEW(beta01); !Define new parameter for covariate effect;

beta01 = exp(b01); !Calculate OR for covariate effect;

NEW(L1prop L2prop); !Define new parameters for class proportions;

L1prop = exp(L1)/(exp(0) + exp(L1)); !Calculate class 1 proportion;

L2prop = exp(0)/(exp(0) + exp(L1)); !Calculate class 2 proportion;

NEW(rho1_1 rho2_1 rho3_1 rho4_1); !Define new parameters for class 1

indicator probabilities;

rho1_1 = (1/(1+exp(c1_1))); !Calculate CRPs for each indicator;

rho2_1 = (1/(1+exp(c2_1)));

rho3_1 = (1/(1+exp(c3_1)));

rho4_1 = (1/(1+exp(c4_1)));

NEW(rho1_2 rho2_2 rho3_2 rho4_2); !Define new parameters for class 1

indicator probabilities;

rho1_2 = (1/(1+exp(c1_2))); !Calculate CRPs for each indicator;

rho2_2 = (1/(1+exp(c2_2)));

rho3_2 = (1/(1+exp(c3_2)));

rho4_2 = (1/(1+exp(c4_2)));

OUTPUT:

TECH1; !List all parameters that are output in the results file;

SAVEDATA:

RESULTS ARE results_1_2_1_1_1_1_1_1.par; !Give filename for parameter

estimates;

SAVE IS CPROBABILITIES; !Save class assignment probabilities in a

separate file;

FILE IS cap_1_2_1_1_1_1_1_1.pro; !Give filename for class assignment

probabilities;
